# Successful Use of Venoarterial Extracorporeal Membrane Oxygenation in Acute Drug Intoxication: Three Cases From a Single Japanese Center

**DOI:** 10.7759/cureus.89451

**Published:** 2025-08-05

**Authors:** Ryo Hokama, Norihiro Goto, Moriaki Shinzato, Hiroyuki Tsuchiya, Kota Hoshino

**Affiliations:** 1 Emergency and Critical Care Center, Okinawa Prefectural Nanbu Medical Center and Children's Medical Center, Haebaru, JPN

**Keywords:** cardiopulmonary support techniques, extracorporeal cardiopulmonary resuscitation, extracorporeal life support, self poisoning, serum lactate

## Abstract

The indications for extracorporeal membrane oxygenation (ECMO) have broadened in clinical practice, and its use in circulatory failure caused by acute drug intoxication has become more frequent. We reviewed three cases of venoarterial (VA) ECMO use for intoxication at our hospital. Three cases (aged 60-69 years) developed refractory shock following intentional overdose, including calcium channel blockers. Despite maximal pharmacologic support, all exhibited progressive hemodynamic deterioration and elevated serum lactate levels (max 8.0-20.0 mmol/L). VA ECMO was initiated on the day of admission in each case. Hemodynamic stability was restored, and ECMO was discontinued within 4 to 6 days. All patients survived hospital discharge without major ECMO-related complications. These cases highlight the potential benefit of the timely initiation of VA ECMO in selected intoxicated patients with drug-induced cardiogenic shock. Comparison with other clinical experiences suggests better outcomes when ECMO is implemented before cardiac arrest. Lactate levels at the time of ECMO initiation may serve as a prognostic indicator. The timely initiation of VA ECMO may be life-saving in patients with acute drug intoxication and refractory cardiogenic shock. Careful patient selection, guided by toxin type and pre-ECMO lactate trends, is essential to optimize outcomes.

## Introduction

Advancements in extracorporeal membrane oxygenation (ECMO) management, circuit biocompatibility, and pump technology have significantly improved patient outcomes and broadened its clinical indications [1]. Originally developed for refractory respiratory failure and cardiogenic shock, ECMO is now being considered in various critical care scenarios, including cardiac arrest, sepsis-induced myocardial dysfunction, and, more recently, toxicologic emergencies.

The reported survival rate of patients receiving ECMO for cardiogenic shock is approximately 62% [2], while venoarterial (VA) ECMO for drug intoxication has demonstrated a survival rate of up to 68.8% [3]. These findings suggest that outcomes for drug-induced cardiogenic shock requiring ECMO support are not necessarily poor and may even be favorable compared to other etiologies of cardiogenic shock.

Although several reports have described the potential efficacy of ECMO for circulatory failure resulting from acute drug intoxication, the Extracorporeal Life Support Organization (ELSO) guidelines currently do not include intoxication as a recommended indication for ECMO [4]. High-quality evidence supporting the use of ECMO remains limited.

In this study, we reviewed three cases of VA ECMO for acute drug intoxication at our institution. This case series did not require approval from the institutional ethics committee, in accordance with the ethical guidelines for medical and health research involving human subjects issued by the Japanese Ministry of Health, Labour and Welfare. All patient data were anonymized in compliance with relevant personal information protection laws. Informed consent for publication was obtained from the patients or their legal guardians.

## Case presentation

Case 1

**Figure 1:**
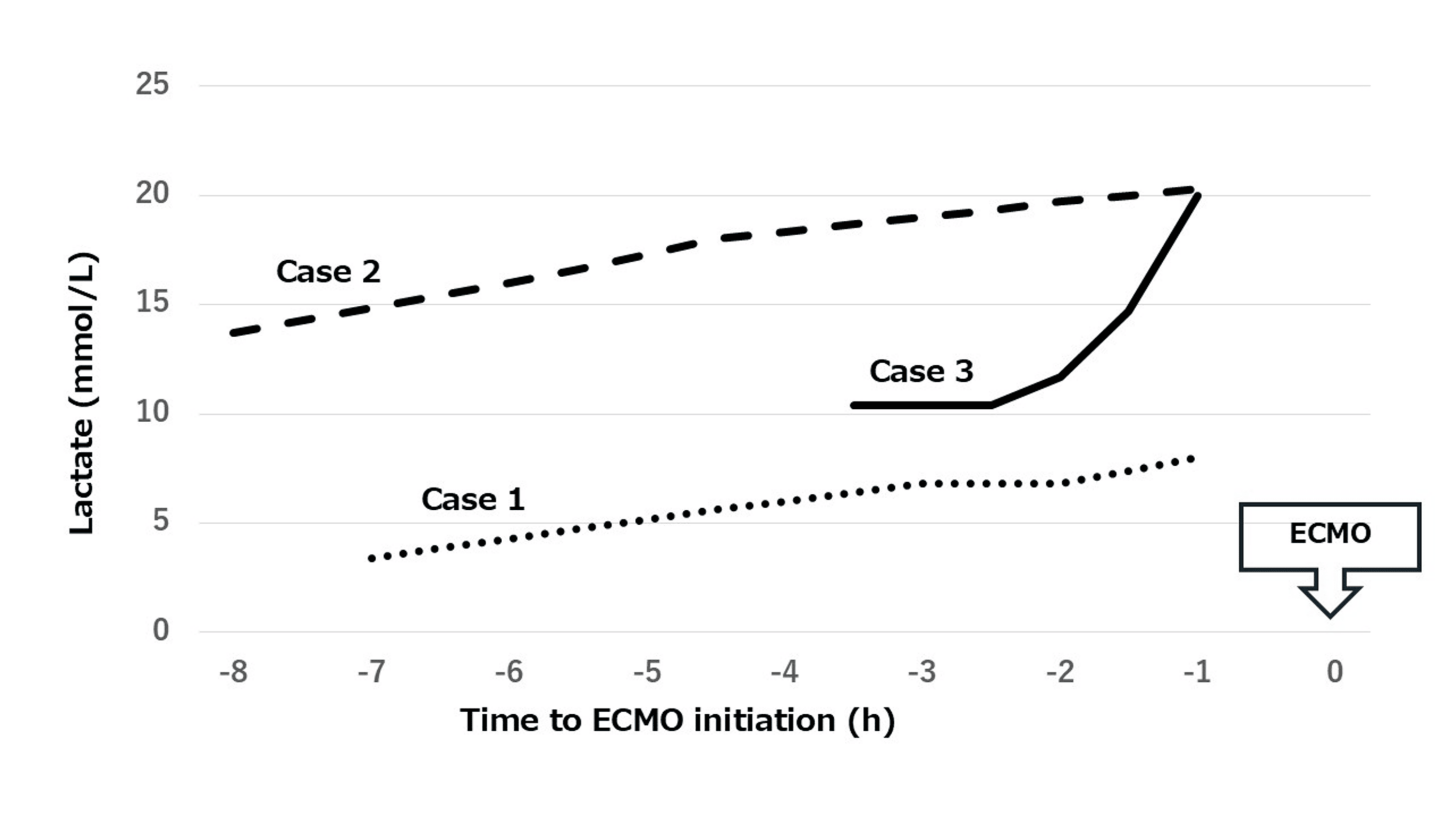
Lactate trends in three cases from our hospital ECMO: extracorporeal membrane oxygenation

A 69-year-old woman was found unconscious. Based on empty blister packs discovered nearby and her prescription records, the ingested medications were suspected to include 600 mg of amlodipine (a calcium channel blocker (CCB)) and 450 mg of diazepam (a benzodiazepine). Activated charcoal was administered for gastrointestinal decontamination. Intravenous calcium preparations and glucagon were administered to counteract CCB toxicity. On arrival, her circulatory status was unstable, with a blood pressure of 60/40 mmHg and a heart rate of 84 bpm. Despite administration of high-dose vasopressors, norepinephrine (0.5 µg/kg/min), dopamine (10 µg/kg/min), and vasopressin (0.03 U/min), her blood pressure remained low at 74/42 mmHg and heart rate at 90 bpm. Serum lactate levels (reference range: 0.5-1.5 mmol/L) continued to rise, peaking at 8.0mmol/L prior to ECMO initiation (Figure 1). VA ECMO was initiated on the day of admission, given the prolonged half-life of amlodipine. Hemodynamic status gradually improved, and ECMO was successfully weaned on day 6. The patient was discharged home on day 54 with a Cerebral Performance Category (CPC) score of 1.

Case 2

A 60-year-old man presented after an overdose of an unknown quantity of antihypertensive and heart failure medications. CCB poisoning was suspected based on the clinical presentation, and calcium preparations along with glucagon were administered. On arrival, his circulatory status was unstable, with a blood pressure of 70/50 mmHg and a heart rate of 62 bpm. Despite high-dose vasopressors, norepinephrine (0.4 µg/kg/min), dopamine (8 µg/kg/min), and vasopressin (0.03 U/min), along with intra-aortic balloon pump support, hypotension persisted with a blood pressure of 56/33 mmHg and a heart rate of 69 bpm. Serum lactate levels continued to rise, peaking at 20.0 mmol/L prior to ECMO initiation (Figure 1). Considering the anticipated lack of spontaneous recovery, VA ECMO was initiated on the day of admission. ECMO was successfully weaned on day 4. The patient was transferred to a rehabilitation hospital on day 54 with a CPC score of 3.

Case 3

A 62-year-old man attempted suicide by ingesting amlodipine (unknown dose), multiple hypnotics, and approximately two liters of glyphosate-based herbicide (Roundup®). Activated charcoal was administered for gastrointestinal decontamination, and calcium preparations and glucagon were given to treat suspected CCB toxicity. On arrival, his blood pressure was 82/62 mmHg and heart rate was 119 bpm. Despite administration of high-dose vasopressors including norepinephrine (1.0 µg/kg/min), hemodynamic instability persisted with a blood pressure of 87/41 mmHg and a heart rate of 90 bpm. Serum lactate levels continued to rise, reaching a peak of 20.0 mmol/L before ECMO initiation (Figure 1). The clinical course was considered to be mainly due to CCB toxicity. Given the expected prolonged toxicity of amlodipine, VA ECMO was initiated. Hemodynamic improvement was observed, and ECMO was successfully discontinued on day 5. He was transferred to a rehabilitation hospital on day 153 with a CPC score of 2.

## Discussion

This study reports three cases in which VA ECMO was implemented for circulatory failure caused by acute drug intoxication. The final diagnosis was CCB-induced cardiogenic shock based on the medication history and hemodynamic instability, despite the absence of confirmatory toxicological analysis. In all cases, calcium chloride and glucagon were administered as antidotal therapies because of a suspected CCB overdose, but therapeutic responses were limited. Despite employing all available detoxification strategies, the lactate levels increased in all cases (Figure 1). VA ECMO was initiated due to the prolonged half-life of long-acting CCBs. Extracorporeal cardiopulmonary resuscitation (ECPR) was not performed for cardiopulmonary arrest in any patient. ECMO support was successfully weaned within 4-6 days, and all three patients were discharged without major ECMO-related complications.

Table 1 provides an overview of six cases of VA ECMO cases for acute drug intoxication: three cases from our institution since 2020 and three additional Japanese cases extracted from Google Scholar using the keywords “intoxication” and “ECMO” (in Japanese) [5-7]. The age distribution included two patients in their 20s, one in their 40s, and three in their 60s. The cohort included three male and three female patients. Four patients had a history of psychiatric disorders, including two with depression, one with bipolar disorder, and one with schizophrenia. Our cases primarily involved CCBs and other pharmaceuticals, whereas the reported cases included various toxic agents, including pharmaceuticals, caffeine (approximately 15 g of anhydrous caffeine), and Aconitum (monkshood). In all reported cases, as well as our cases, ECMO was initiated on the day of admission. In Case 5, the lactate level before ECMO initiation was the highest (22 mmol/L), and VA ECMO was used for ECPR following cardiac arrest. Among these six cases, five patients survived with ECMO support. Two patients presented with lethal arrhythmias, and only one case requiring ECPR after cardiac arrest resulted in mortality, whereas all five patients with pre-arrest ECMO initiation survived.

**Table 1:** Data of six cases with acute poisoning PMH: past medical history; BP: blood pressure; HR: heart rate; CHDF: continuous hemodiafiltration; ECPR: extracorporeal cardiopulmonary resuscitation; ECMO: extracorporeal membrane oxygenation; CCB: calcium channel blocker; BZP: benzodiazepine; ARB: angiotensin II receptor blocker

No.	First Author	Age (years)	Sex	PMH	Type of drugs	BP (mmHg)	HR (bpm)	Noradrenalin (μg/kg/min)	CHDF	Life-threatening arrhythmia	ECPR	pH level before ECMO	Lactate level before ECMO (mmol/L)	Duration of ECMO (days)	Prognosis
1	Hokama	69	F	Depression	CCB, BZP	55/33	86	0.25	+	-	-	7.206	8	6	Survival
2	Hokama	60	M	None	CCB, β-blocker, ARB, Aspirin, Alcohol, etc.	49/30	49	0.4	+	-	-	7.246	20	4	Survival
3	Hokama	62	M	Bipolar disorder	CCB, Pesticide, etc.	58/40	84	0.5	+	-	-	7.009	20	6	Survival
4	Sekine [5]	40s	F	Depression	Pilsicainide, etc.	68/40	60	Unknown dose	-	-	-	7.528	1.7	2	Survival
5	Kano [6]	20s	F	Schizophrenia	Caffeine	0	0	0	+	+	+	7.015	22	2	Death
6	Ueno [7]	20	M	Unknown	Aconite, Alcohol	64/48	150	0.1	-	+	-	7.42	5.3	3	Survival

Patients requiring ECMO for circulatory failure due to acute drug intoxication are rare. According to a U.S. database analysis from 2010 to 2013, of 26,271 cases of drug intoxication, only 10 received ECMO [8]. Another study conducted from 2014 to 2020 found that of 10,771 ICU admissions for drug intoxication, 22 (including 12 ECPR cases) underwent VA ECMO [9]. Although ECMO use for drug intoxication remains infrequent, its utilization appears to have increased over time [10].

In 2023, the American Heart Association (AHA) released guidelines on resuscitation management for acute drug intoxication, recommending early VA ECMO initiation for refractory cardiogenic shock or lethal arrhythmias despite antidote administration [11]. However, ECMO efficacy varies by toxin type, and indications should be carefully considered based on the ingested substance. Drugs such as CCBs and β-blockers can cause severe hypotension and bradycardia, leading to fatal cardiogenic shock. In addition, certain antiarrhythmic and antipsychotic medications are also known to provoke lethal arrhythmias. VA ECMO appears to be beneficial for drug toxicity. By contrast, survival rates were significantly lower for intoxications affecting metabolism or the hematologic system (49% vs. 72%; P=0.004) [10]. Furthermore, ECMO is ineffective against toxins that cause direct cellular toxicity (e.g., chemotherapeutic agents, heavy metals, and paraquat) or disrupt cellular oxygen utilization (e.g., carbon monoxide and cyanide) [11].

CCB overdose induces profound cardiogenic shock through inhibition of L-type calcium channels, leading to decreased myocardial contractility, bradycardia, and peripheral vasodilation. Long-acting agents such as amlodipine have prolonged half-lives (30-50 hours) and high lipid solubility, resulting in delayed clearance and prolonged toxicity. Standard therapies, including calcium infusion, glucagon, vasopressors, and high-dose insulin, may be insufficient in severe cases. VA ECMO provides temporary circulatory support, maintaining perfusion and oxygenation while the toxin is metabolized. Its role is particularly critical in reversible drug-induced shock, where it can serve as a bridge to recovery. Given the pharmacokinetics of CCBs and the expected reversibility with time, the use of ECMO in the management of amlodipine poisoning is both rational and pathophysiologically justified.

In a study of 62 patients with acute drug intoxication resulting in cardiac arrest and/or severe cardiogenic shock, the ECMO group had a significantly higher survival rate than the conservative treatment group [86% (12/14) vs. 48% (23/48); P<0.05] [12]. Additionally, a review of 64 cases who received VA ECMO for drug intoxication identified arterial blood pH as an independent prognostic predictor [13]. Another study of 22 cases who received VA ECMO found that lactate levels before ECMO initiation were a prognostic factor, with a threshold value of 9 mmol/L for predicting in-hospital mortality (AUC: 0.916, 95% CI: 0.79-1.04, P=0.001) [9]. Notably, the only fatal case in our study had the highest lactate level (22 mmol/L) prior to ECMO initiation. Although ECMO can adsorb lipophilic drugs onto the artificial lung membrane and tubing [14,15], its potential effect on reducing drug concentrations was not evaluated in our study. Currently, ECMO should be considered primarily for circulatory support rather than for detoxification.

For acute drug intoxication, primary treatments such as gastric lavage, activated charcoal, antidotes, and hemodialysis/adsorption should be prioritized. Temporary VA ECMO support may be warranted in cases of refractory cardiogenic shock or lethal arrhythmias [16]. While early ECMO deployment based on toxin type and lactate trends before cardiac arrest may improve outcomes, caution is necessary. Given the limited sample size, these findings should be considered hypothesis-generating rather than conclusive. The decision to initiate ECMO must be individualized, taking into account the specific toxin involved and the reversibility of the underlying pathophysiology. Multicenter studies are warranted to further clarify appropriate indications and timing of ECMO in cases of acute intoxication.

## Conclusions

Among three cases of VA ECMO for acute drug intoxication, all cases survived until discharge. In cases of refractory drug intoxication, ECMO initiation, based on the type of toxin and trends in lactate levels, may be beneficial. Timely VA ECMO initiation may be critical in managing acute drug intoxication with circulatory failure.
